# The TEMP-PREVENT trial: a study protocol for a randomized, double-blind, placebo-controlled clinical trial of etidronate for treatment in young adult patients with pseudoxanthoma elasticum

**DOI:** 10.1186/s13063-025-09064-6

**Published:** 2025-10-23

**Authors:** Iris M. Harmsen, Melanie Haverkamp, Tim C. van den Beukel, Madeleine Kok, Pim A. de Jong, Evelien van Valen, Redmer van Leeuwen, Jeannette Ossewaarde-van Norel, Wilko Spiering

**Affiliations:** 1 Department of Vascular Medicine, University Medical Center Utrecht, Utrecht University, P.O. Box 85500, Utrecht, 3508 GA the Netherlands; 2 Department of Radiology, University Medical Center Utrecht, Utrecht University, Utrecht, the Netherlands; 3 Department of Ophthalmology, University Medical Center Utrecht, Utrecht University, Utrecht, the Netherlands; 4 Department of Neuropsychology, University Medical Center Utrecht, Utrecht University, Utrecht, the Netherlands

**Keywords:** Pseudoxanthoma elasticum, Etidronate, Randomized controlled trial, Arterial calcifications

## Abstract

**Background:**

Pseudoxanthoma elasticum (PXE) is a genetic disease with autosomal recessive inheritance, characterized by slowly progressive ectopic calcification in the elastic fibers of the skin, Bruch’s membrane in the retina, and in the arteries. Etidronate was found to be effective in halting the progression of arterial calcification in an earlier trial, including middle-aged patients with PXE with manifest arterial disease. It is uncertain if there are similar effects in young adult patients with PXE.

**Methods:**

The TEMP-PREVENT study is a randomized, double-blind, placebo-controlled clinical trial, investigating the effect of 20 mg/kg cyclical etidronate on ectopic calcification in PXE patients of ≥ 18 and ≤ 55 years. The study is conducted at the University Medical Center, Utrecht, the Netherlands. The primary endpoint is the change in arterial calcification volume between baseline and 24 months in the leg arteries and carotid siphons, as measured on low-dose unenhanced Computed Tomography. Secondary endpoints are changes in dermatological, ophthalmological, vascular, laboratory, cognitive and quality of life outcomes/parameters. Recruiting started in April 2023. Results are expected in 2027 and will be dispersed through peer-reviewed journals and international conferences.

**Discussion:**

PXE is a rare genetic disease for which there is currently no registered treatment. The TEMP-PREVENT trial is set out to evaluate the effects of etidronate in young adult patients with PXE.

**Trial registration:**

The trial is registered at the Dutch Central Committee on Research Involving Human Subjects (CCMO) (registration number: NL75350.041.21), European Union Clinical Trials Register (EudraCT number: 2021–000434-34), and at ClinicalTrials.gov (ID: NCT05832580, registration date 2023-04-14).

## Introduction

### Background and rationale

Pseudoxanthoma elasticum (PXE) is an autosomal recessive genetic disorder characterized by slowly progressive ectopic calcification in the elastic fibers of the skin, Bruch’s membrane in the retina, and the arteries. The exact prevalence is unknown, but is estimated to be 1 in 25,000–50,000 [1–4]. Clinical manifestations cause considerable morbidity, with ocular events leading to legal blindness, skin lesions developing in early infancy, and vascular complications resulting in peripheral artery disease, gastric bleeding, and increased occurrence of cerebrovascular events [5–7]. Currently, there is no registered treatment for PXE. 

PXE is caused by pathogenic variants in the *ABCC6* gene [8]. The exact role of the *ABCC6* transporter is not fully elucidated, but the generally accepted hypothesis is that the *ABCC6* protein acts as an ATP transmembrane transporter in the liver and kidneys [9]. Because ATP is metabolized to ADP and inorganic pyrophosphate (PPi), the absence of functional *ABCC6* in PXE leads to reduced plasma concentrations of PPi, a potent calcification inhibitor [10–13].

Bisphosphonates are chemically stable analogues of PPi [13]. Bisphosphonates have been widely used for over 40 years to treat conditions such as osteoporosis and have proven to be remarkably safe ^14,15^. The molecular structure of the different bisphosphonates results in either a strong osteoclast-inhibiting potential or more affinity to bind to hydroxyapatite [14, 15]. This makes etidronate particularly interesting in the treatment of PXE, because it has a relatively low impact on osteoclast activity, and a large impact on inhibition of mineralization [16].

A proof-of-concept study demonstrated that etidronate reduced soft tissue calcification in an *ABCC6* -/- mice model [17]. This finding was confirmed in a randomized, double-blind, placebo-controlled clinical trial in PXE patients [18]. After 1 year of treatment, arterial calcification had decreased by 4% (IQR, − 11% to 7%) in the etidronate group and increased 8% (IQR, − 1% to 20%) in the placebo group [18, 19]. Arterial calcification was, however, not a primary endpoint. Moreover, the trial included only PXE patients with extensive arterial calcification, resulting in a relatively older and more affected population. It is unclear if arterial calcification is equally inhibited in a young adult population with fewer PXE manifestations. Moreover, etidronate only inhibits the growth of calcification and does not reverse it, so prevention of further ectopic calcification early on may be even more beneficial than inhibiting it at a late stage. Another benefit of a young trial population is that calcification of Bruch’s membrane is better visible in eyes that show no gross structural damage to the macula, such as choroidal neovascularization or pigment epithelium or retinal atrophy, as often happens in later stages of PXE.

Therefore, in the TEMP-PREVENT trial, we aim to determine the effect of etidronate treatment in PXE patients with minimal or no existing arterial calcification on the development and progression of arterial calcification, and also on ocular calcification in Bruch’s membrane, dermatological calcifications, and cardiovascular outcomes.

### Trial design

The TEMP-PREVENT trial (an acronym for “Treatment of Ectopic Mineralization in Pseudoxanthoma elasticum-PREVENTion”) is a randomized, double-blind, placebo-controlled clinical trial comparing the effects of 24 months of treatment with etidronate versus placebo in patients with PXE aged 18 to 55.

## Methods

### Study setting

The study will take place at the UMC Utrecht Expertise Center for Pseudoxanthoma elasticum (UECP) in Utrecht, the Netherlands. This is the Dutch National Expertise Center, in which most Dutch patients with PXE are seen. Patient recruitment started in April 2023. This study has received funding from the Dioraphte Foundation, the Dutch PXE Foundation (Stichting PXE-Fonds), the Dutch Eye Foundation (de Oogvereniging), and the Dutch Eye Society (het Oogfonds). The trial is registered at the Dutch Central Committee on Research Involving Human Subjects (CCMO) (registration number: NL75350.041.21), European Union Clinical Trials Register (EudraCT number: 2021–000434-34), and at ClinicalTrials.gov (ID: NCT05832580). This protocol was developed in accordance with the SPIRIT reporting guidelines.

### Study population

Patients will be recruited from the UECP located in the University Medical Center Utrecht (UMCU) and by recruitment through (social) media, for example, using the UMCU website, the Dutch PXE Society, newsletters, yearly patient days, and conferences. In total, 76 patients will be recruited. To be eligible to participate in this study, a subject must meet the following inclusion criteria: (1) age between 18 and 55 years, (2) definitive or probable diagnosis of PXE according to the Plomp criteria [20], and (3) fertile women should take adequate contraceptive measures. Reasons for exclusion are the following: (1) patients unable or unwilling to sign for informed consent, (2) for women: pregnant, lactating, or fertile and wish to become pregnant within 3 years, (3) estimated glomerular filtration rate below 30 ml/min/1.73m^2^ according to the CKD-EPI equation [21], (4) known abnormality of the esophagus that would interfere with passage of the drug (e.g., esophagus stenosis), (5) chronic diarrhea (> 1/month), (6) known osteomalacia, (7) hypocalcemia (calcium < 2.20 mmol/L corrected for albumin), (8) vitamin D deficiency (< 35 nmol/L), (9) use of bisphosphonate in the last 5 years, (10) known sensitivity to etidronate, (11) any other medical or social condition that, at the discretion of the Principal Investigator, might put the subject at risk of harm during the study or might adversely affect the interpretation of the study data. The eligibility screening will be performed by the treating physician. Individuals withdrawn from the study (for any reason) will be replaced.

### Informed consent

All patients who fit the criteria for inclusion will be contacted by phone by their treating physician. During this call, study procedures will be briefly explained to the subject, and if the patient is interested in participation, an envelope with the patient’s information folder (PIF), including an informed consent form, will be sent to the subject. After the patient has received and read the contents of this envelope, the patient will again be called and, if still interested, invited for a screening appointment. There will be at least 10 days between sending the PIF and this second call to allow potential participants enough time to consider their decision. During the screening appointment in the UMCU, the patient’s understanding of the study and its procedures will be verified, and an informed consent form will be signed before any procedures by the patients and research physician. The informed consent procedure will be executed by a trained research nurse or physician.

### Intervention

Patients will be randomly assigned to either daily, oral etidronate capsules of 200 mg or 400 mg at 20 mg/kg for 2 weeks, followed by 10 weeks off (9 cycles in total), *or* an identical product without the active pharmacological substance (i.e., a placebo) in the same cyclical regimen, for a total of 24 months. The choice of a placebo control group was made with careful consideration and is made because no proven effective treatment exists for PXE.

Etidronate capsules are administered orally, as oral intake is the safest, convenient, and cost-effective mode of administration. Etidronate is best absorbed on an empty stomach. Patients will be thoroughly explained how to ingest the product: on an empty stomach, in an upright position, and are instructed to remain in an upright position for 30 min. Subjects are advised to refrain from simultaneous intake of calcium-containing products to improve absorption. The total dose per patient is calculated based on their body weight and then rounded to the nearest dose divisible by 400 mg.

At the initial product dispensing and each follow-up visit, an adherence reminder session will take place. At follow-up visits, patients will be asked about any problems they have with taking their study medication. Also, any reasons for missed doses will be addressed. Moreover, patients will have the opportunity to ask questions, and the importance of following our study guidelines will be emphasized. Patients can continue to take medications for other conditions as normal. The sponsor/investigator has liability insurance, which is in accordance with Article 7 of the WMO. The sponsor also has insurance, which is in accordance with the legal requirements in the Netherlands (Article 7 WMO). This insurance provides cover for damage to research subjects through injury or death caused by the study.

There is already forty years of experience with etidronate, and the side-effect profile is well-known and relatively mild. The most common side effects are gastrointestinal complaints, including loose stools, abdominal discomfort, and nausea [22]. A rare, but serious adverse effect of bisphosphonates is osteonecrosis of the jaw. This is, however, only reported in patients with intravenous bisphosphonate use, mostly when treating cancer-related hypercalcemia ^22^. Theoretically, etidronate could have an effect on calcium homeostasis and bone turnover. Therefore, measurements of plasma calcium, phosphate, and vitamin D will be included as safety measures [23].

Etidronate and the matching placebo will be produced by Pharmacy A15, Gorinchem, the Netherlands. The products will be packaged and shipped to the Department of Clinical Pharmacy of the UMCU. At every follow-up visit, participants will be asked to take any redundant product and the empty packaging material to monitor drug adherence.

## Outcome measures

The primary endpoint in the TEMP-PREVENT trial is the percentage change in arterial calcification volume in the carotid siphons and leg arteries, measured on low-dose (< 3 ms for a 70 kg adult), unenhanced, thinslice, multi-detector CT scan (CT7500, Philips Healthcare, Cleveland, Ohio). Tube voltage (kVp) is set at 120, and tube current (mAs) varies between patients, reliant on body weight. Arterial calcification volume in microliter (µL) will be measured at a threshold of 130 Hounsfield Units using commercially available software (Calcium Scoring Heartbeat, Philips Advanced Visualization Workspace, Philips Healthcare).

Secondary endpoints are changes in dermatological, ophthalmological, vascular, laboratory, cognitive, and quality of life parameters. *Dermatological* outcomes are changes in elastin degradation and calcification in skin biopsies. A 3-mm punch biopsy will be taken from non-lesional skin, at least 2 cm from a pseudoxanthoma lesion, on the back of the neck just below the hairline, and under local anaesthesia. To ensure standardization and repeatability, a photograph of the biopsy site will be taken, and at the 24-month follow-up (M24), a second biopsy will be performed at least 2 cm from the original site to avoid scar tissue interference. The extent of elastin degradation will be scored on Verhoeff-Van Gieson stains, and the extent of calcification will be scored on Von Kossa stains [24]. All analyses will be performed by an experienced dermatopathologist. *Ophthalmological* outcomes are changes in best-corrected visual acuity and low luminance visual acuity, contrast sensitivity, changes in the infrared images, autofluorescence patterns, and the appearance of new choroidal neovascularisations on optical coherence tomography (OCT)-angiography, Bruch’s membrane reflectivity on high-definition spectral domain OCT, and the number of anti-VEGF injections. OCT is a non-invasive imaging technique requiring mydriatic eyedrops [25]. *Vascular* outcomes are changes in carotid intima-media thickness (cIMT) by ultrasound, which is a non-invasive measurement to establish the carotid atherosclerotic vascular disease and measures the thickness of the tunica intima and media of the carotid artery [26]. Moreover, changes in pulse wave velocity [27] and pulse wave analysis [28] are measured to reflect changes in arterial stiffness. Ankle-brachial index [29], the six-minute walking test [30], and the WELCH questionnaire [31, 32] are compared between baseline and 24 months of follow-up to reflect peripheral arterial disease. Moreover, changes in the pulsatility index of the intracranial carotid arteries and middle cerebral arteries by 7T-MRI are compared. The incidence of major adverse cardiovascular events (TIA, stroke, myocardial infarction, and/or cardiovascular death) is counted to see if etidronate lowers the risk of these events. *Laboratory* outcomes are changes in calcium, phosphate, ASAT, ALAT, eGFR, desmosin, and inorganic pyrophosphate [33]. *Cognitive outcomes* are changes in memory, attention, and executive function. Memory will be assessed with the Rey auditory verbal learning test with immediate recall, delayed recall, and recognition [34]. Attention will be tested with the Trail Making Test-A [35], Stroop Test reading and colour naming [36], and Digit Span Forwards WAIS-III [37]. Executive function will be tested with the Trail Making Test-B [35], Stroop Test colour-word Interference [36], Digit Span Backwards WAIS-III [37], Semantic fluency [38], and the Brixton Test [39]. Composite Z-scores will be calculated for the different cognitive domains using normative data from the Advanced Diagnostics Infrastructure (ANDI) [40]. The differences in change in cognitive functioning between baseline and follow-up will be compared between the intervention group and the control gro*up. Quality of life* outcomes are changes in EQ-5D [41], PROMIS 10 [42], PROMIS PF [43], and USER P [44].

## Participant timeline

Abbreviations: *CT* computed tomography, *VA* visual acuity, *CS* contrast sensitivity, *IR* infrared, *FAF* fundus autofluorecence, *OCTA* optical coherence tomography angiography, *SD-OCT* spectral domain optical coherence tomography, *anti-VGEF* anit vascular endothelial growth factor, *MRI* magnetic resonance imaging, *cIMT* carotid Intima media thickness, *PWV* pulse wave velocity, *ABI* ankle brachial index, *ASAT* aspartate aminotransferase, *ALAT* alanine aminotransferase (Table 1).

**Table 1:** Schedule of study procedures and visits

Time:		M0	M6	M12	M18	M24
Visit:	1 (screening)	1		2		3
Eligibility screening	X					
Informed consent	X					
Low dose CT of legs and head (siphons)		X				X
Dermatological measurements						
Skin biopsy		X				X
Ophthalmological measurements						
Measurement of VA and CS		X				X
Fundus imaging (IR, FAF and OCTA)		X				X
SD-OCT scanning		X		X		X
No. of anti-VEGF injections		X	X	X	X	X
Vascular measurements						
Cerebral MRI		X				X
cIMT, PWV, PWA, and ABI		X				X
6 min walk test		X				X
WELCH questionnaire		X				X
Laboratory measurements						
Plasma Pyrophosphate		X				X
Desmosine		X				X
Laboratory measurements for safety monitoring (Calcium, 25-OH vitamin D, Creatinine, Phosphate, Magnesium, Albumin, ASAT, ALAT)	X			X		X
EQ-5D		X				X
PROMIS 10		X				X
PROMIS PF		X				X
USER P		X				X

*CT* computed tomography, *VA *visual acuity, *CS *contrast sensitivity, *IR* infrared, *FAF *fundus autofluorecence, *OCTA* optical coherence tomography angiography, *SD-OCT* spectral domain optical coherence tomography, *anti-VGEF* anit vascular endothelial growth factor, *MRI* Magnetic resonance imaging, *cIMT* carotid Intima media thickness, *PWV* pulse wave velocity, *ABI* ankle brachial index, *ASAT* aspartate aminotransferase, *ALAT* alanine aminotransferase

## Sample size calculation

Sample size calculations for the TEMP-PREVENT trial were performed using the results of the TEMP trial [19]. The TEMP trial showed a relative difference in mean siphon and leg calcification of 14% between placebo and control for 1 year of treatment. We anticipate calcification to increase in a non-linear manner over the course of the life of a PXE patient. Therefore, for the sample size calculation, we assumed a lower relative difference of 10% over 2 years. With this difference, a power of 80% and a two-sided alpha of 0.05, we would need 33 subjects per arm. With an expected dropout rate of 15%, we would need 38 subjects per arm, resulting in 76 subjects for the trial. All statistical analyses will be performed using R, R Foundation for Statistical Computing, Vienna, Austria.

## Assignment of interventions

### Allocation

Randomisation will be performed 1:1 using computer-generated block randomization with variable block sizes by the Clinical Drug Research Unit of the UMCU. Participants and researchers are both blinded to treatment allocation. Participants are enrolled by the research physician. The research physician is not involved in the randomisation procedure.

### Blinding

Patients and the study team will be blinded to treatment allocation. The study visits will be conducted by the research physician who is blinded to treatment allocation. The deblinding list will be kept in the Department of Clinical Pharmacy. Deblinding of study medication of individual participants is only possible in the case of a medical emergency, in which deblinding has consequences for the medical treatment of the participant.

## Data collection

An overview of visits is shown in Table 1. At baseline, patients visit the UMCU, and after taking the screening for eligibility and signing of informed consent, study data is collected. Patients undergo a CT scan, skin biopsy, ophthalmologic measurements plus retinal imaging, carotid ultrasound, pulse wave analysis, ankle brachial index measurement, 6-min walking test, a 7T-MRI of the brain, cognitive testing, and laboratory evaluation, all on the same day. After the first visit, questionnaires are sent out via e-mail. At 6 and 18 months, the patients are called to evaluate the number of anti-VEGF injections used or if any adverse events have occurred. At 12 months, patients visit the UMCU again for laboratory testing and retinal OCT scanning. At 24 months, all testing performed at baseline is repeated. Participants may withdraw from the study for any reason at any time. Early discontinuation of the study medication is not a reason for withdrawal from the study, and follow-up visits will be continued as scheduled. 

For data collection, e-CRFs are used in Electronic Data Capture of Castor (Amsterdam, the Netherlands). Digital pseudonymized files are stored within the UMCU Research Folder Structure. Independent external party Julius Clinical will conduct data monitoring. All data will be handled confidentially. Study information will not be released outside of the study without the written permission of the participant, except as necessary for monitoring. Subject data will be stored using the subject identification codes. The key to the codes will be kept by the researcher and the data manager. The subject identification code list will be treated confidentially at all times, and by the GDPR.

## Statistical analysis

Descriptive data will be presented as numbers and percentages for categorical data, mean and standard deviations for normally distributed continuous data, or median and interquartile ranges for non-normally distributed continuous data. Data will be analyzed applying the intention-to-treat principle. When missing data occurs, potential patterns will be analyzed and appropriate methods used accordingly (i.e., complete case analyses or multiple imputation) [44]. Researchers will be blinded during statistical analysis. The mean change (delta) from baseline to 24 months will be calculated for both groups for all primary and secondary endpoints. These deltas will be analyzed with a two-tailed Student’s *t*-test or Mann–Whitney *U* test as appropriate. In the case of relevant baseline differences, adjustments will be made with regression models. Both adjusted and unadjusted models will be presented. Also, if the numbers are sufficient, subgroup analyses will be performed in patients with different PXE phenotypes (predominantly ophthalmological phenotype, predominantly dermatological phenotype, predominantly vascular phenotype).

## Oversight and monitoring

### Data monitoring

Given the low-risk profile of this trial and in line with EMA guidelines, an external data safety monitoring board is not required. However, due to the potential side effects of etidronate, safety will be periodically reviewed by an independent data safety monitoring board (DSMB). Interim safety analyses will be conducted at 6, 12, and 18 months, evaluating laboratory parameters and ophthalmological data (anti-VEGF injections). The interim analysis is performed by an independent statistician, blinded to the treatment allocation. The statistician will have unblinded access to all data and will report the results of the analysis to the independent DSMB. The DSMB, including at least a professor in biostatistics and a medical specialist, will discuss the results of the interim analysis and will issue binding safety recommendations to the ethics committee.

### Harms

Adverse events are defined as any undesirable experience occurring to a subject during the study, whether or not considered to be related to the investigational product (etidronate). All adverse events reported spontaneously by the subject or observed by the investigator or his staff will be recorded. An adverse event that meets the criteria for a serious adverse event (SAE) will be reported to the local ethics committee as an SAE. A SAE for this study is any untoward medical occurrence or effect that, whether or not considered or not be related to the investigational product that results in death, is life threatening, requires hospitalization or prolongation of existing hospitalization, results in persistent or significant disability or incapacity, is a congenital anomaly or birth defect or any other important medical event that, due to medical or surgical intervention, did not result in any of the outcomes listed above, but could have based upon appropriate judgement by the investigator. An elective hospital admission will not be considered a serious adverse event.

### Audit and monitoring

No independent audits are planned for this study. Oversight will be ensured through routine monitoring activities conducted by the TSF. These monitor visits aim to ensure protocol compliance, verify informed consent procedures, assess source data verification, and evaluate data quality and completeness. The TSF monitors will conduct their visits independently of the study investigators and sponsor. Any deviations or issues identified during monitoring will be documented and addressed in collaboration with the study team. Escalation to the sponsor or ethics committee will occur if required.

### Ethical considerations

The study will be conducted according to the principles of the Declaration of Helsinki (Fortaleza, Brazil, October 2013) and in accordance with the Dutch Medical Research Involving Human Subjects Act (WMO), the requirements of Good Clinical Practice (GCP), and this study protocol (Version 7.0, 2024). The study is approved by the Medical Ethical Review Committee, NedMec (registration number 21–552), as well as the CCMO. Any modifications to the protocol that may impact the conduct of the study, the potential benefit of the patient, or may affect patient safety, including changes of study objectives, study design, patient population, sample sizes, study procedures, or significant administrative aspects, will require a formal amendment to the protocol. Such an amendment will be agreed upon by the study team and approved by the Ethics Committee before implementation.

## Results

Participant recruitment commenced in April 2023, with anticipated results to be available in 2027. Findings will be shared through peer-reviewed publications and presentations at both national and international conferences.

## Discussion

The TEMP-PREVENT trial will provide insight into the effect of 24 months of treatment with etidronate in young adult patients with PXE.

Previously, the TEMP-trial has shown that a year of etidronate treatment in PXE patients gave a reduction in arterial calcification of 4% (interquartile range [IQR], − 11% to 7%) when compared to placebo. This trial did, however, exclusively enroll patients with measurable arterial calcification, comprising, therefore a relatively older and more profoundly affected study population. It remains uncertain to what extent arterial calcification can be halted in a young adult population. 

A young adult study population has the additional advantage that the retina is not yet, or at least to a lesser degree, affected by choroidal neovascularization and atrophy [45]. The TEMP trial showed a difference in neovascularizations between the etidronate (1) and placebo group (9); however, post hoc analysis showed this was likely not an effect of etidronate but could be explained by the unequal distribution of patients more at risk of CNV activity among the two groups [46]. Using newly developed techniques in measuring reflectivity of Bruch’s membrane in patients without extensive structural damage of the macula, the aim is to better study the effect of etidronate on ophthalmologic manifestations in PXE patients.

## Trial status

The protocol version number and date are: Version 7.0, 24/06/2024. The recruitment of the first participant started on April 26, 2023. The last patient was recruited on December 4, 2024. The last patient visit of the study is scheduled for December 2026.

## Data Availability

Data can be made available upon request. The study team had access to the final trial dataset. There are no contractual agreements that limit access for our investigators.

## Abbreviations

CCMO: Dutch Central Committee on Research Involving Human Subjects
DSMB: Data safety monitoring board
GCP: Good clinical practice
PPi: Inorganic pyrophosphate
PXE: Pseudoxanthoma elasticum
SAE: Serious adverse event
UECP: UMC Utrecht Expertise Center for Pseudoxanthoma elasticum
UMCU: University Medical Center Utrecht
WMO: Dutch Medical Research Involving Human Subjects Act
